# *Mahayograj guggulu*: Heavy metal estimation and safety studies

**DOI:** 10.4103/0974-7788.72486

**Published:** 2010

**Authors:** G. S. Lavekar, B. Ravishankar, S. Gaidhani, V. J. Shukla, B. K. Ashok, M. M. Padhi

**Affiliations:** *CCRAS, 61-65, Institutional Area, Opposite “D” Block, Janakpuri, New Delhi, India*; 1 *Department of Pharmacology, IPGT&RA, Gujarat Ayurved University, Jamnagar, India*

**Keywords:** Heavy metal content, *Mahayograj guggulu*, safety, toxicity

## Abstract

**Objective:**

This study was conducted to estimate the heavy metal profile and determine the safety of *Mahayograj guggulu*, an Ayurvedic herbo-mineral preparation.

**Design:**

*Mahayograj guggulu*, manufactured by Shree Baidynath Ayurved Bhawan Pvt. Ltd., Gwalior Road, Jhansi - 284 003 (of batch number-07 and manufacturing date October 2004) was procured from the local market. Heavy metal concentrations were measured using an atomic absorption spectrophotometer. A total of 40 Charles Foster strain albino rats of either sex with an average body weight of 160–250 g were divided into four groups (Groups I, II, III and IV), with 10 animals in each group. Group I served as the control, while Group II, III and IV rats received *Mahayograj guggulu* at a dose of 54 (dose equivalent to human therapeutic dose), 270 (five-times the dose equivalent to the human therapeutic dose) and 540 (10-times the dose equivalent to human therapeutic dose) mg/kg, p.o. for 120 days. The effect of drug administration was noted on the ponderal, biochemical, hematological and histopathological parameters. In addition, urine examination was also carried out. At the end of the study, only six rats per group were sacrificed as per the IAEC advice.

**Results:**

*Mahayograj guggulu* was found to be safe at all dose levels tested. No significant behavioral changes were noted in any of the groups studied. The effect on food and water consumption and fecal and urine output remained unaffected in all groups during the study period. No major alterations were observed in hematology, serum biochemistry, necropsy and histopathology at the therapeutically advocated dose level. Heavy metal content measurement indicated levels of 25.8 µg/g for lead, 0.07 µg/g for mercury and 5.19 µg/g for arsenic.

**Conclusions:**

The test drug is well tolerated as no changes of a serious nature could be observed in any of the parameters assessed.

## INTRODUCTION

*Rasaushadhi* is an integral part of Ayurveda, and describes the use of metals and minerals for chronic disorders in various combinations, dosage forms and at various levels of purities. The recent concern raised about safety by certain researchers from other fields about these classical metal-based preparations has led to an attempt to revalidate the safety of these medicines (which have been in use in India for centuries) in a more rigorous way, to improve the global acceptance.

The safety and efficacy of a herbomineral depend on the complex methodology adopted for its preparation, and any deviation from the “classical” (i.e., as described in the texts of Ayurveda) preparation method will not yield desired results.

*Mahayogaraj guggulu*,[1] an Ayurvedic proprietary (classical) medicine and one among those listed by Saper *et al*.[1,2] as having an unacceptably huge metallic content, has been in use in Ayurveda for the treatment of various neurological disorders.[1,3] This study was conducted to assess the toxicity of this agent in rats in order to validate the claims of Ayurvedic texts regarding its safety.

## MATERIALS AND METHODS

### 

#### Mahayograj guggulu

*Mahayograj guggulu* (batch number-07 and manufacturing date October 2004) manufactured by Shree Baidynath Ayurved Bhawan Pvt. Ltd. with the batch number and date of preparation as identified by Saper *et al*.[1] was procured from the local market. *Mahayograj guggulu*[2,3] contains several plants and metals that are listed in Table 1.

**Table 1:** Ingredients of *Mahayograj guggulu*

Name of the ingredient	Sanskrit name	Part used	Content
*Zingiber officinale* Rosc.	Nagara (Sunthi)	Rz.	3 g
*Piper longum* Linn.	Pippali	Fr.	3 g
*Piper longum* Linn.	Pippali mula	Rt.	3 g
*Piper rectrofractum* Vahl.	Chavya	St.	3 g
*Plumbago zeylanica* Linn.	Chitraka	Root	3 g
*Ferula narthexs* Bioss.	Hinga bharta	Exd	3 g
*Trachyspermum ammi* (L.) Sprague	Ajamoda	Fr.	3 g
*Brassica campestris* Linn.	Sarsapa	Sd.	3 g
*Cuminum cyminum* Linn.	Sweta jiraka	Fr.	3 g
*Carum carvi* Linn.	Krisna jiraka	Fr.	3 g
*Vitex negundo* Linn.	Renuka	Sd	3 g
*Holarrhena antidysenterica* (Roxb.ex Flem.) Wall	Indrayava (Kutja)	Sd.	3 g
*Cissmpelos pareira* Linn. Hirsute (DC.) Forman	Patha	Root	3 g
*Embelia ribes Burn*. f.	Vidanga	Fr	3 g
*Scindapsis officinalis* (Roxb.) Schott.	Gajapippali	Fr	3 g
*Picrorhiza kurroa* Royle ex Benth.	Katuka	Rt/Rz	3 g
*Aconitum heterophyllum* Wall.	Ativisa	Rt/tr	3 g
*Clerodendrum serratum* (Linn.) Moon	Bharangi	Rt	3 g
*Acorus calamus* Linn.	Vacha	Rz	3 g
*Marsdenia tenacissima* (Roxb.) Moon	Murva	Rt	3 g
*Terminalia chebula* Retz.	Haritaki	P	40 g
*Terminalia bellirica* (Gaertn.) Roxb.	Bibhitaki	P	40 g
*Phyllantnus emblica* Linn.	Amalaki	P	40 g
*Commiphora wightii* (Arn.) Bhandari.	Guggulu-shodhita	Ext.	180 g
Incinerated metals			
Tin	Vanga Bhasma	-	48 g
Silver	Rajat Bhasma	-	48 g
Lead	Naga Bhasma	-	48 g
Iron	Loha Bhasma	-	48 g
Mica	Abhraka Bhasma	-	48 g
Iron oxide	Mandura Bhasma	-	48 g
Mercuric sulfide	Rasa Sindura (Parada)	-	48 g

The drug was dispensed in plastic bags and labelled with an alphabetic identifier, and was dispatched to the Pharmacology Research Unit, Jamnagar, for toxicity studies without interruption of the custody chain. Research personnel were blinded to the identity of the drug. The following procedures were adopted for the different studies:

### Estimation of heavy metals

The samples were weighed separately and analyzed for lead (Pb), cadmium (Cd), arsenic (As) and mercury (Hg) by atomic absorption spectrophotometry.[4]

### Toxicity studies

Charles Foster albino rats of either sex with body weight ranging from 160 to 180 g were obtained from the animal house facility of the Institute of Post Graduate Teaching and Research in Ayurveda (IPGTRA), Gujarat Ayurveda University, Jamnagar, Gujarat. They were maintained under appropriate laboratory conditions in the prevailing ambient temperature (22 ± 3°C) and humidity (50–70%) conditions. The experiments were carried out in accordance with the guidelines of the Institute’s Animal Ethics Committee after obtaining its permission. The dose for experimentation was calculated with reference to the suggested human doses, i.e. 600 mg/day.[5] The animals were divided into four groups of 10 animals each.

Chronic toxicity was conducted using a single, daily oral administration of the test drug at 54 (therapeutic dose), 270 (five-times the therapeutic dose) and 540 (10-times the therapeutic dose) mg/kg for 120 days. The test drug was given as a suspension in distilled water by gavage, with the control group receiving the vehicle (distilled water).

Toxicity was evaluated by observing the effects of the test drug on the body weight and gross behavioral changes. Biochemical variables like blood sugar,[6] serum total cholesterol,[7] serum triglyceride,[8] serum urea,[9] serum creatinine,[10] serum alkaline phosphatase (ALP),[11] SGOT (Serum glutamic oxaloacetic transaminase)[12] and SGPT (Serum glutamic pyruvic transaminase) activity,[13] total protein,[14] serum albumin and serum globulin[15] were estimated using an autoanalyzer (ERBA CHEM-5, Trans Asia) using standard kits available at the end of the study. Hematological parameters like WBC, RBC, platelet count, lymphocyte percentage, MCV(mean corpuscular volume,), monocyte percentage, hematocrit, granulocyte percentage, MCH (mean corpuscular haemoglobin), MCHC (mean corpuscular hemoglobin concentration), lymphocyte count, MRBC (macro red blood cells) and hemoglobin were carried out using an auto-cell counter (MS-9 Veterinary Melet Schloesing hematology cell counter, France).

The qualitative analysis of urine was performed with respect to the estimation of sodium and potassium, pH of urine following standard procedures[16] and urine microscopy were also carried out.

At the end of the treatment, only six of the 10 animals (as per the advice of the ethics committee) were sacrificed and gross and histological appearance of vital organs (brain, pituitary, thymus, lymph node, heart, lungs, liver, stomach, spleen, kidney, testis, uterus, bone marrow and ovary) were examined.[17] Bone marrow smears were also prepared.

### Statistical method

The data were subjected to statistical analysis using Student’s “t”-test.[18]

## RESULTS

### Estimation of heavy metals in the drug

Heavy metal content determined using an atomic absorption spectrophotometer are presented in Table 2. The corresponding values in our study are 25.800 µg/g for lead, 0.07 µg/g for mercury and 5.19 µg/g for arsenic. The comparison shows that the values reported by Saper *et al*.[2] are higher in comparison with the values reported by us. Our estimation shows that the values for lead alone are higher than those prescribed by WHO. The other values are within the prescribed limits.

**Table 2:** Heavy metal estimation in the Ayurvedic formulation, *Mahayograj guggulu*

Name of the organization	Pb (10 ppm)*	Hg (1 ppm)*	As (10 ppm)*	Cd (0.3 ppm)*
CCRAS†	25.800 µg/g	0.07 µg/g	5.19 µg/g	0.94 µg/g
JAMA††	37.000 µg/g	22.800 µg/g	8.100 µg/g	-----

* Limits of heavy metals (WHO),

† Technique used for the estimation – atomic absorption spectrophotometer,

†† Technique used for the estimation – X-ray fluorescence spectroscopy

### Toxicity studies

No significant behavioral changes were observed in any of the groups studied. During the study, five of the 40 animals (one from the control group on the 36^th^ day, three from therapeutically equivalent dose [TED] × 5 group on the 7^th^, 18^th^ and 24^th^ day and one from TED × 10-treated group on the 16^th^ day). Necropsy revealed no evidence of toxicity. Effect on food and water consumption and fecal and urine output remained unaffected in all groups during the study period.

Comparable body weight gain was observed in the control as well as the test drug-administered groups. The body weight gain was slightly higher in the TED and the TED × 5 groups. A marginal decrease in weight gain was observed in the TED × 10 group [Table 3]. The observed difference was found to be statistically non-significant.

**Table 3:** Dose-dependent effect of *Mahayograj guggulu* on body weight (120 days) in albino rats

Groups	Dosage (mg/kg)	Body weight (g)		Body weight change (g)	Body weight change (%)
		Initial	At the end of the study		
Control	Distilled water	205.00 ± 9.57	255.83 19.76	50.83 ± 15.83	----
TED	54	194.83 ± 7.61	253.66 ± 15.34	60.50 ± 12.92	19.02↑
TED × 5	270	221.66 ± 11.66	277.66 ± 28.45	56.00 ± 20.59	10.17↑
TED × 10	540	231.66 ± 15.14	276.66 ± 26.66	48.33 ± 18.15	4.91↑

Data: mean ± SEM, ↑, increase; ↓, decrease

A moderate decrease in the weight of the thymus was observed in the TED and the TED × 5-treated groups, while the test drug administration did not affect the weight of the thymus in the TED × 10-treated group to a significant extent in comparison with the control group. The test drug administration did not affect the liver weight to a significant level at dose levels of TED × 5 and TED × 10. A marginal decrease was observed in the spleen weight in the TED dose-treated group in comparison with the control group; however, the observed decrease was statistically non-significant [Table 4].

**Table 4:** Effect of different dose levels of *Mahayograj guggulu* on the organ weight (mg) of albino rats

Name of organ	Control (distilled water)	TED (54 mg/kg)	TED × 5 (270 mg/kg)	TED × 10 (540 mg/kg)
Thymus	586.33 ± 46.66	484.16 ± 25.70	475.50 ± 34.51	605.66 ± 29.27
Heart	766.33 ± 43.98	720.00 ± 36.05	1003.83 ± 204.96	796.83 ± 59.37
Liver	6300.00 ± 405.77	5355.66 ± 275.49	6395.50 ± 552.41	7036.66 ± 681.15
Spleen	535.00 ± 48.38	487.50 ± 28.86	615.50 ± 66.47	650.00 ± 60.06
Kidney	1383.33 ± 116.15	1448.33 ± 063.94	1683.83 ± 173.78	1670.66 ± 161.78
Testis	2880.00 ± 69.28	2358.33 ± 182.76*	2893.33 ± 246.05	2703.00 ± 110.24

Data: mean ± SEM,

* P < 0.05

The data related to the effect of test drug on different biochemical parameters are presented in Table 5. A marginal decrease observed in the blood glucose and serum cholesterol levels in the test drug-administered group was found to be statistically non-significant in comparison with the vehicle control group. The test drug administration did not affect serum triglyceride and blood urea levels to a significant extent as compared with the control group. An apparent decrease in the serum creatinine level was observed in the test drug-administered groups in comparison with the control group, and this was found to be statistically non-significant. All three test drug-treated groups showed a statistically significant increase in the ALP activity and a non-significant increase in SGOT in comparison with the control group. This was not dose dependent. In the TED group, a marginal increase in serum globulin was observed in comparison with the control group. A moderate decrease in the serum globulin level was observed in the TED × 5-treated groups. A statistically significant decrease in the serum globulin level was observed in the TED × 10-treated group compared with the control group [Table 5].

**Table 5:** Effect of different dose levels of *Mahayograj guggulu* on the biochemical parameters in albino rats

Parameter studied	Control (distilled water)	TED (54 mg/kg)	TED × 5 (270 mg/kg)	TED × 10 (540 mg/kg)
Blood glucose (mg/dl)	78.83 ± 3.67	66.50 ± 4.66	75.83 ± 3.36	69.33 ± 3.65
Serum cholesterol (mg/dl)	58.83 ± 7.09	64.33 ± 7.84	66.66 ± 3.42	63.00 ± 3.07
Serum triglycerides (mg/dl)	117.50 ± 15.53	104.33 ± 16.26	124.33 ± 17.95	113.50 ± 6.34
Blood urea (mg/dl)	38.00 ± 3.25	34.83 ± 1.62	36.83 ± 2.89	39.00 ± 2.73
Serum creatinine (mg/dl)	0.98 ± 0.07	0.90 ± 0.03	00.86 ± 0.04	00.93 ± 0.06
Serum alkaline phosphatase (IU/L)	137.67 ± 32.66	296.00 ± 31.62**	266.66 ± 25.04**	251.16 ± 33.36*
SGOT (IU/L)	276.67 ± 20.77	356.00 ± 41.84	288.66 ± 42.25	428.60 ± 88.86
SGPT (IU/L)	89.00 ± 6.82	107.00 ± 27.25	084.50 ± 4.67	102.20 ± 9.77
Serum total protein (g/dl)	7.43 ± 0.40	7.65 ± 0.26	07.10 ± 0.15	07.08 ± 0.13
Serum albumin (g/dl)	3.55 ± 0.32	3.53 ± 0.22	03.80 ± 0.26	04.08 ± 0.10
Serum globulin (g/dl)	3.80 ± 0.16	4.11 ± 0.39	03.30 ± 0.28	03.00 ± 0.11**

Data: mean ± SEM,

* *P* < 0.05,

** *P* < 0.01

All the test drug-treated groups showed a decrease in the WBC count in comparison with the control group. However, only the decrease observed in the TED × 5 group was found to be statistically significant in comparison with the control group. An apparent decrease in the lymphocyte count was observed at all the three dose levels studied, although only the decrease observed at the TED × 5 dose level was found to be statistically significant in comparison with the control group. The test drug showed a marginal decrease in the hemoglobin level in the test drug-treated groups; however, this observed decrease was found to be statistically non-significant [Table 6]. Although a decrease in macro-RBC was observed, it had no pathological significance as their count increases in megaloblastic type of anemia. A decrease in the number of macro-RBCs is indicative of RBC formation of uniform size, which is a desired response. The histopathology of the bone marrow revealed normal profile and cellularity [Table 6]. The data related to the effect of test drug on pH, sodium and potassium content of the urine are depicted in Table 7. The test drug at the dose levels studied did not affect any of these parameters to significant extent.

**Table 6:** Effect of different dose levels of *Mahayograj guggulu* on the hematological parameters in albino rats

Parameters	Control (distilled water)	TED (54 mg/kg)	TED × 5 (270 mg/kg)	TED × 10 (540 mg/kg)
WBC (10 e3/µl)	1.404 ± 0.16	1.170 ± 0.18	0.920 ± 0.13*	1.070 ± 0.26
RBC (10 e6/µl)	6.634 ± 0.68	5.810 ± 0.31	5.610 ± 0.23	6.150 ± 0.31
Lymphocyte%	96.76 ± 0.52	95.05 ± 1.04	95.58 ± 1.21	93.25 ± 2.14
Monocyte%	1.28 ± 0.11	1.16 ± 0.22	1.20 ± 0.27	1.58 ± 0.14
Granulocyte%	1.94 ± 0.42	3.75 ± 0.90	3.21 ± 0.99	3.28 ± 0.80
Lymphocyte count (10 e3/µl)	1.36 ± 0.17	1.12 ± 0.17	0.88 0.13*	1.10 ± 0.25
MCV (fl)	70.96 ± 2.16	75.05 ± 1.59	68.98 ± 1.27	67.25 ± 1.11
Hematocrit (%)	46.52 ± 3.27	43.40 ± 1.60	38.83 ± 2.16	41.31 ± 2.05
MCH (pg)	16.54 ± 3.14	16.71 ± 0.85	14.91 ± 0.36	14.36 ± 0.30
MCHC	16.54 ± 3.14	16.71 ± 0.85	14.91 ± 0.36	14.36 ± 0.30
MRBC%	0.82 ± 0.08	0.76 ± 0.08	0.43 ± 0.14*	0.51 ± 0.10*
Hemoglobin (g)	10.46 ± 1.36	9.60 ± 0.22	8.41 ± 0.46	8.85 ± 0.45

Data: mean ± SEM,

* *P* < 0.05

**Table 7:** Effect of different dose levels of *Mahayograj guggulu* on urine pH sodium and potassium excretion in albino rats

Groups	Dosage (mg/kg)	Urine pH	Sodium excretion (mEq/I)	Potassium excretion (mEq/I)
Control	Distilled water	9.00 ± 0.00	26.10 ± 5.02	81.00 ± 22.99
TED	54	8.83 ± 0.16	23.20 ± 5.80	74.69 ± 26.61
TED × 5	270	9.00 ± 0.00	22.04 ± 6.46	72.48 ± 30.28
TED × 10	540	9.00 ± 0.00	72.52 ± 30.29	106.59 ± 34.77

Data: mean ± SEM

**Figure 1:**
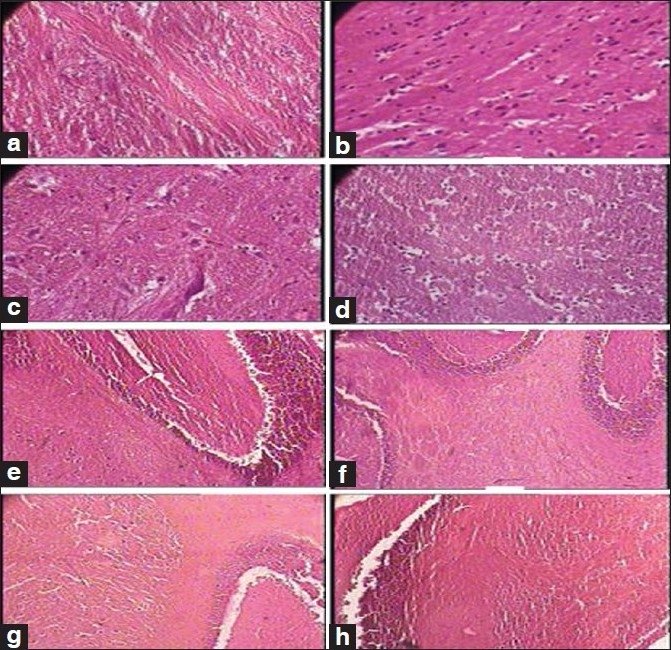
Photomicrographs of sections of hindbrain and cerebellum Note the normal cytoarchitecture in all the groups, (a): Hindbrain of the control group (1 × 400 magnification), (b, c): Hindbrain of the therapeutically equivalent dose group (1 × 400 magnification), (d): Hindbrain of the therapeutically equivalent dose × 10 group (1 × 400 magnification), (e): Cerebellum of the control group (1 × 100 magnification), (f): Cerebellum of the therapeutically equivalent dose group (1 × 100 magnification), (g): Cerebellum of the therapeutically equivalent dose × 5 group (1 × 100 magnification), (h): Cerebellum of the therapeutically equivalent dose × 10 group (1 × 100 magnification)

**Figure 2:**
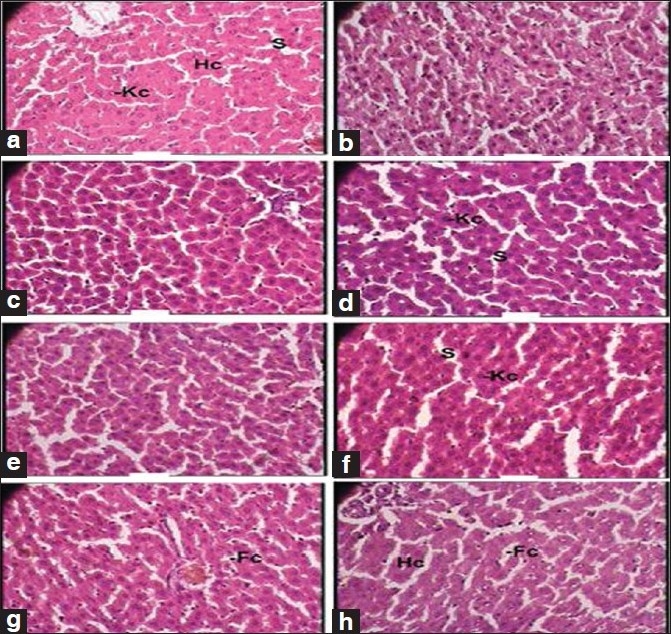
Photomicrographs of sections of the liver. Note the normal cytoarchitecture, (a, b): Control group (1 × 400 magnification). Hc, hepatic cell; Kc, Kupffer cell; S, sinusoid, (c, d): Therapeutically equivalent dose group (1 × 400 magnification). Kc, Kupffer cell; S, sinusoid, (e, f): Therapeutically equivalent dose × 5 group (1 × 400 magnification). Kc, Kupffer cell; S, sinusoid, (g, h): Therapeutically equivalent dose × 10 group (1 × 400 magnification). Hc, hepatic cell

**Figure 3:**
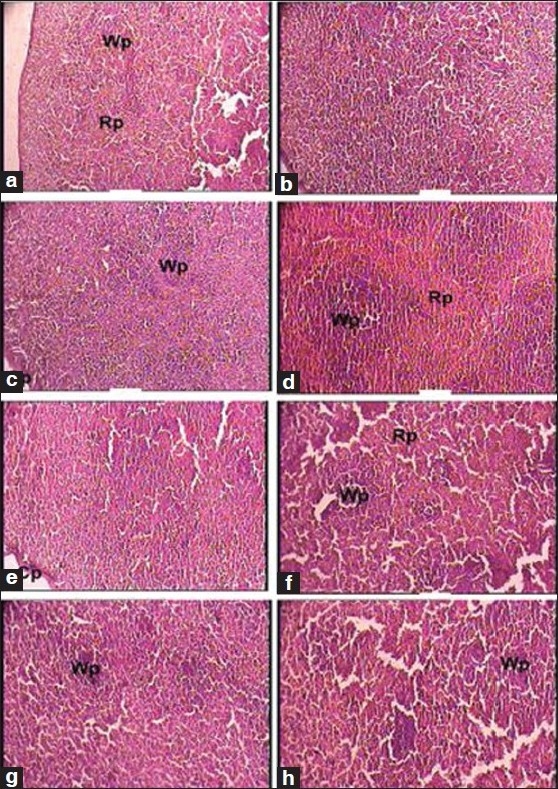
Photomicrographs of sections of the spleen. Note: (a) Normal cytoarchitecture in a, b, c and e. (b) Increased white pulp in d, f, g and h, (a, b): Control group (1 × 100 magnification). Rp, red pulp; Wp, white pulp, (c): Therapeutically equivalent dose group (1 × 100 magnification). Cp, capsule; Wp, white pulp, (d) Therapeutically equivalent dose group (1 × 100 magnification). Rp, red pulp; Wp, white pulp, (e): Therapeutically equivalent dose × 5 group (1 × 100 magnification). Cp, capsule, (f–h): Therapeutically equivalent dose × 10 group (1 × 100 magnification). Rp, red pulp; Wp, white pulp

**Figure 4:**
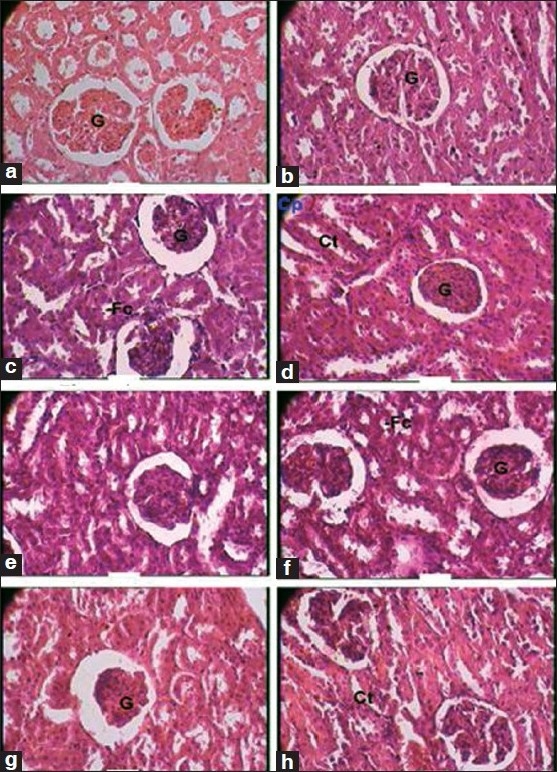
Photomicrographs of sections of the kidney Note the normal cytoarchitecture, (a, b): Control group (1 × 400 magnification). Cp, capsule; Ct, convoluted tubule; G, glomerulus, (c): Therapeutically equivalent dose group (1 × 400 magnification). G, glomerulus, (d): Therapeutically equivalent dose group (1 × 400 magnification). Cp, capsule; Ct, convoluted tubule; G, glomerulus, (e, f): Therapeutically equivalent dose × 5 group (1 × 400 magnification). Cp, capsule; Ct, convoluted tubule; G, glomerulus, (g, h): Therapeutically equivalent dose × 10 group (1 × 400 magnification). Ct, convoluted tubule; G, glomerulus

**Figure 5:**
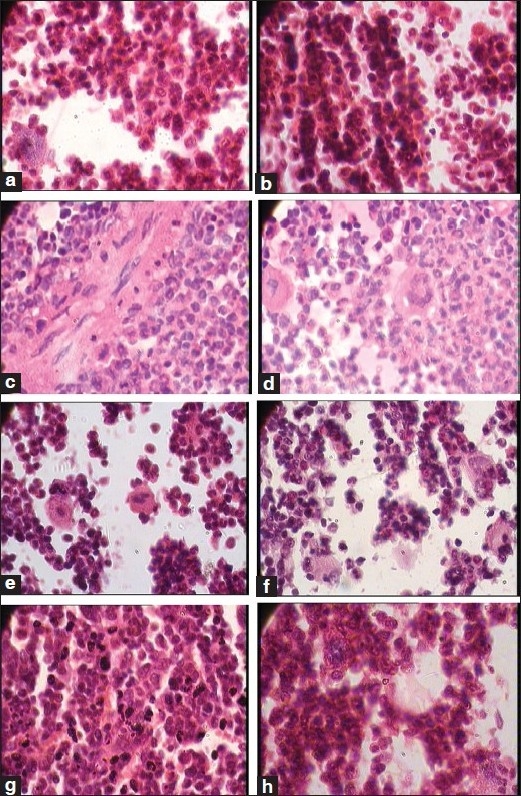
Photomicrographs of sections of bone marrow, (a, b): Control group (1 × 1,200 magnification), (c, d): Therapeutically equivalent dose group (1 × 1,200 magnification), (e, f): Therapeutically equivalent dose × 5 group (1 × 1,200 magnification), (g, h): Therapeutically equivalent dose × 10 group (1 × 1,200 magnification)

**Figure 6:**
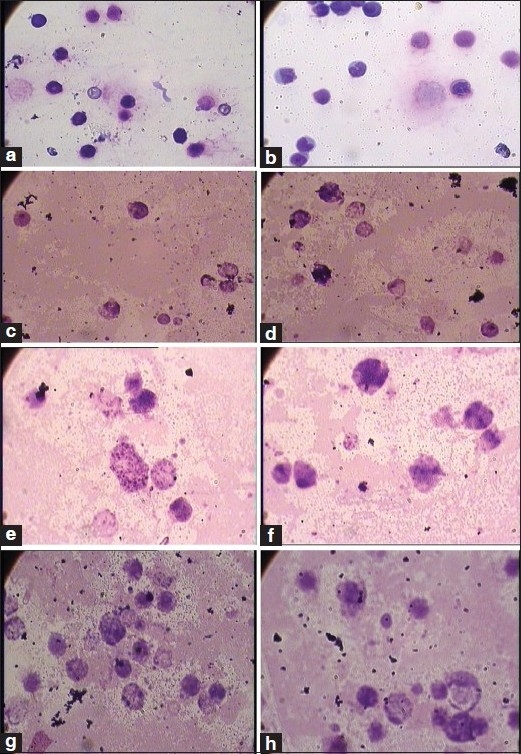
Photomicrographs of the bone marrow smear, (a, b): Control group (1 × 1,200 magnification), (c, d): Therapeutically equivalent dose group (1 × 1,200 magnification), (e, f): Therapeutically equivalent dose × 5 group (1 × 1,200 magnification), (g, h): Therapeutically equivalent dose × 10 group (1 × 1,200 magnification)

**Figure 7:**
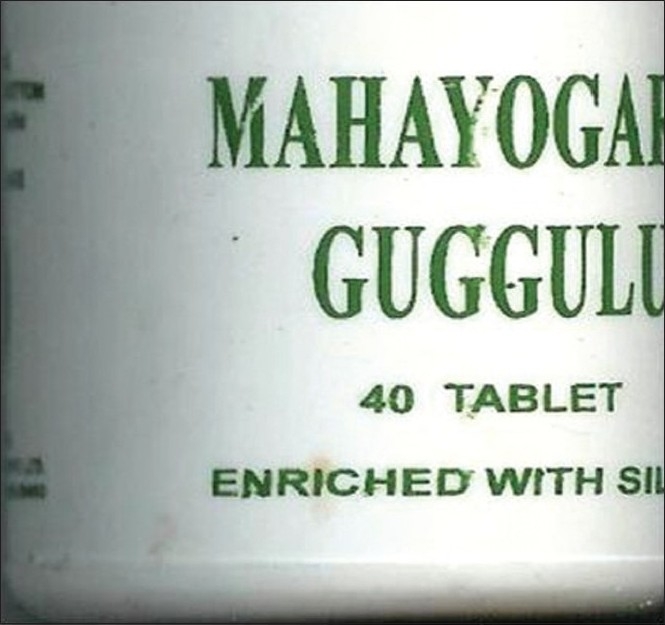
Photograph of *Mahayogaraj guggulu*

**Figure 8:**
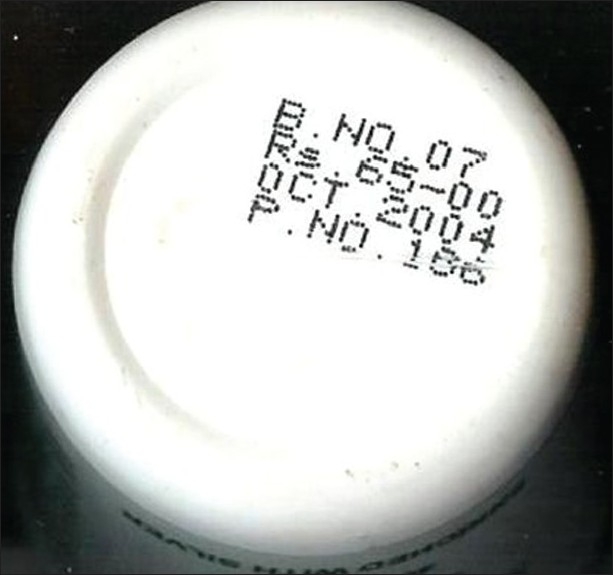
Photograph of *Mahayogaraj guggulu* showing batch number

Besides this, the test drug did not produce any significant changes in the cytoarchitecture of any organ studied at any of the dose levels [Figures 1–8]. None of the organs showed any pathological changes except the spleen, which showed an increase in the white pulp proportion in some rats, and this was not considered to have any pathological significance.

## DISCUSSION

The Ayurvedic drug *Mahayograj guggulu* manufactured as per the classical method was tested for its content of heavy metals and chronic toxicity.

As compared with the heavy metal contents reported by Saper *et al*.[2] in the same batch of the drug, we found different amounts of heavy metals (lead: 37 µg/g as against 25.8 µg/g in our study; mercury: 22.8 µg/g as against 0.07 µg/g in our study; arsenic: 8.1 µg/g as against 5.19 µg/g in our study). The reason for this variation could be the method of determination of the levels of the metal. Saper *et al*.[2] used the X-ray diffraction analysis, which, by its non-destructive nature, may give higher values than those that actually exist.

Body weight change is an important index for the assessment of toxicity. In the present study, the test drug, even at the highest dose level studied, showed almost normal body weight gain. This clearly indicates that it does not cause serious organ damage or derange any physiological function.

Of the 13 biochemical parameters studied, significant changes were observed in the ALP activity at all dose levels. This enzyme is synthesized in the liver and bone. It is produced by the osteoblast of the bone and is associated with the calcification process. It is localized in the cell membranes and is associated with the transport mechanism in the liver, kidney and intestinal mucosa. Moderate (two to three-times) increase in the ALP level is seen in hepatic diseases such as infective hepatitis, alcoholic hepatitis or hepatocellular carcinoma.[19] Because corroborative changes in histopathological study could not be seen in the liver, kidney and heart, the involvement of these organs may be ruled out. It is possible that the observed effect may be due to the increased osteoblast activity. Moderate elevation was observed in the serum total bilirubin level, although this was also not dose dependent and could have resulted from a higher destruction of the RBCs. However, hematological investigations show only a marginal change in the RBC count and marginal to moderate decrease in the hemoglobin content. Taking these factors into consideration, it may be inferred that the elevations do not indicate any serious pathology. A decrease in the serum globulin was observed at higher dose levels. This parameter is influenced by many factors due to its varied components. Because no degenerative changes could be observed in the thymus, spleen and lymph node, immunological toxicity can be ruled out.

A decrease was noted in the total WBC count. This decrease may be indicative of suppression of the formation of WBCs. However, as the effect was not dose dependent and not seen at a therapeutically equivalent dose, it may not be of any serious therapeutic concern. Changes observed in the RBC and platelet-related parameters were not remarkable.

No significant change in the cytoarchitecture of the important organs studied could be observed. This clearly indicates that the formulation has no serious toxicological implications.

A higher level of lead is reported to produce neurologic, immunological, reproductive and renal toxicity.[20] In the present study, no behavioral or neurological toxicity was observed in the behavioral studies. Because the spleen, thymus and lymph node were not affected structurally, serious adverse effects on the immune system do not seem to be involved. However, functional effects cannot be ruled out as no cytokine assay was carried out and a fall in the WBC count was observed. Similarly, the kidney and reproductive organs from the test drug-administered groups did not exhibit any degenerative change on histological examination. Further, the biochemical markers related to the kidney and liver were not affected.

In spite of the presence of lead in levels more than the permitted limits, serious lead toxicity did not occur. The only observation that was seen included fall in WBC counts and elevation of ALP, for which there was no other corroborating clinical, biochemical or histopathological explanation. There could be several explanations for this. As this formulation is herbomineral in nature, there is a possibility of an interaction between the metal and plant component during preparation, which might lead to a decreased bioavailability of the metal. Secondly, the lead may not be present purely in an inorganic form. Formation of organometallic complexes may also, hypothetically, influence the bioavailability of lead. Further, the formulation contains other metal-based products, which may lead to a metal–metal interaction, modulating the bioavailability of the other.

Thus, the administration of the herbomineral formulation presents a highly complex biological situation that cannot be explained simply on the basis of simple measurements of the heavy metal content in them. Even the toxicokinetic study with tissue estimation of the metal contents may not clarify the issue unless the speciation of the metal in the biological system can be established. Thus, the only reliable indicator of safety of these preparations is assessing their effect in biological systems. In the present study, this was carried out in the form of a chronic toxicity study (120 days). In spite such a long duration of administration, no serious toxic effects could be observed, especially at doses equivalent to the clinically advocated dose.

In conclusion, this study indicated that *Mahayograj guggulu* is generally well tolerated. The only possible cause for concern was the moderate elevation of ALP activity and moderate decrease in the total WBC and lymphocyte count and the tendency toward a decreased platelet count, all of which suggest myelosuppression. Importantly, none of the effects observed were dose dependent in nature, and a histopathological examination showed an almost normal cytoarchitecture of the organs studied, ruling out a serious toxicity potential at the therapeutic dose levels.
